# Colon Cancer Presenting as an Incarcerated Incisional Hernia: A Case Report

**DOI:** 10.7759/cureus.103576

**Published:** 2026-02-14

**Authors:** Masayuki Nakamura, Yujo Kawashita, Koya Umeda, Teruo Nakaya, Masaki Tateishi, Masashi Haraguchi, Takashi Ueda, Junzo Yamaguchi, Seiko Harada, Sosei Abe, Yoichi Hachitanda

**Affiliations:** 1 Surgery, Fukuoka Seishukai Hospital, Fukuoka, JPN; 2 Gastroenterology, Fukuoka Seishukai Hospital, Fukuoka, JPN; 3 Pathology, Fukuoka Seishukai Hospital, Fukuoka, JPN

**Keywords:** adenocarcinoma of colon, general emergency surgery, incarcerated incisional hernia, incisional ventral hernia, post cholecystectomy, right-sided colon cancer

## Abstract

Colorectal cancer within a hernia sac is rare. Although cases involving inguinal hernias have been reported, colorectal cancer in an incisional hernia has not been documented. A 91-year-old woman with a history of cholecystectomy 30 years earlier presented with melena, hematemesis, and anorexia. CT showed an incisional hernia at the right subcostal region containing the ascending colon with wall thickening and fat stranding, raising concern for incarceration and possible malignancy. Emergency laparotomy revealed an incarcerated ascending colon with a type 2 adenocarcinoma. Right hemicolectomy and primary hernia repair were performed. Histopathology confirmed pT4aN0M0 adenocarcinoma with venous congestion but no tumor rupture or worsening of lymphovascular invasion. The patient was discharged on postoperative day 22. This appears to be the first reported case of colorectal cancer in an incisional hernia. Occult malignancy should be considered in elderly patients with complicated incisional hernias, and early surgery is advisable for irreducible cases.

## Introduction

Colorectal cancer ranks as the third most prevalent malignancy worldwide and remains a significant surgical challenge [1]. The disease predominantly affects elderly populations, with the median age at diagnosis exceeding 70 years, making management decisions particularly complex in this demographic [1].

Incisional hernias represent a common complication following abdominal surgery, occurring in 10%-20% of patients after laparotomy [2]. These hernias typically contain the small bowel or omentum, and their management ranges from elective repair to emergency intervention when complications such as incarceration or strangulation develop.

Colorectal cancer presenting within a hernia sac is uncommon. Since the first description in 1938 [3], colorectal cancer in inguinal hernias has been reported sporadically, with approximately 40 cases documented in the literature [4-6]. In these cases, the sigmoid colon is most frequently involved (71%-82%), while ascending colon cancer accounts for fewer than 6% of cases [4]. The clinical presentation is often acute: among reported cases, 47% present with incarceration, 41% present with perforation, and only 6% are discovered incidentally [5]. This high rate of complicated presentations underscores the diagnostic challenge and the importance of maintaining clinical suspicion for malignancy in elderly patients with hernias.

Despite these reports involving inguinal hernias, colorectal cancer within an incisional hernia has not been documented. To the best of our knowledge, this is the first report of colon adenocarcinoma incarcerated in an incisional hernia, occurring 30 years after cholecystectomy.

## Case presentation

A 91-year-old Japanese woman presented with a one-month history of anorexia, black emesis, and melena. Her medical history included hypertension, type 2 diabetes mellitus, and an overactive bladder. She had undergone a total hysterectomy in her 20s and an open cholecystectomy via a right subcostal incision at age 60. Two weeks earlier, she had been treated for bilateral lower extremity cellulitis with ceftriaxone for 12 days.

On examination, vital signs were stable. A firm, non-tender, irreducible mass was palpable at the right subcostal incision site. The abdomen was otherwise soft without peritoneal signs. Laboratory findings showed moderate anemia (hemoglobin: 9.0 g/dL), hypoalbuminemia (2.2 g/dL), elevated inflammatory markers (C-reactive protein {CRP}, 12.92 mg/dL; white blood cell {WBC}, 10,802/μL), and mildly prolonged prothrombin time (PT)-international normalized ratio (INR) (1.28). Tumor markers were within normal limits (carcinoembryonic antigen {CEA}, 2.3 ng/mL; carbohydrate antigen 19-9 {CA19-9}, 36.9 U/mL) (Table 1).

**Table 1:** Laboratory findings on admission. AST, aspartate aminotransferase; ALT, alanine aminotransferase; PT-INR, prothrombin time-international normalized ratio; APTT, activated partial thromboplastin time; CEA, carcinoembryonic antigen; CA19-9, carbohydrate antigen 19-9; GOT, glutamic oxaloacetic transaminase; GPT, glutamic pyruvic transaminase

Parameters	Value	Reference Range
Hematology		
White blood cell count	10,802/μL	3,300-9,000
Hemoglobin	9.0 g/dL	11.5-15.0
Hematocrit	27.0%	35.0-45.0
Platelet count	25.0 × 10⁴/μL	15.0-35.0
Biochemistry		
Total protein	5.0 g/dL	6.5-8.2
Albumin	2.2 g/dL	3.8-5.2
Blood urea nitrogen	16.3 mg/dL	8.0-20.0
Creatinine	0.68 mg/dL	0.40-0.80
AST (GOT)	12 U/L	10-40
ALT (GPT)	8 U/L	5-45
Total bilirubin	0.77 mg/dL	0.2-1.2
C-reactive protein	12.92 mg/dL	<0.30
Coagulation		
PT-INR	1.28	0.90-1.10
APTT	35.1 seconds	24.0-39.0
Tumor markers		
CEA	2.3 ng/mL	<5.0
CA19-9	36.9 U/mL	<37

**Figure 1:**
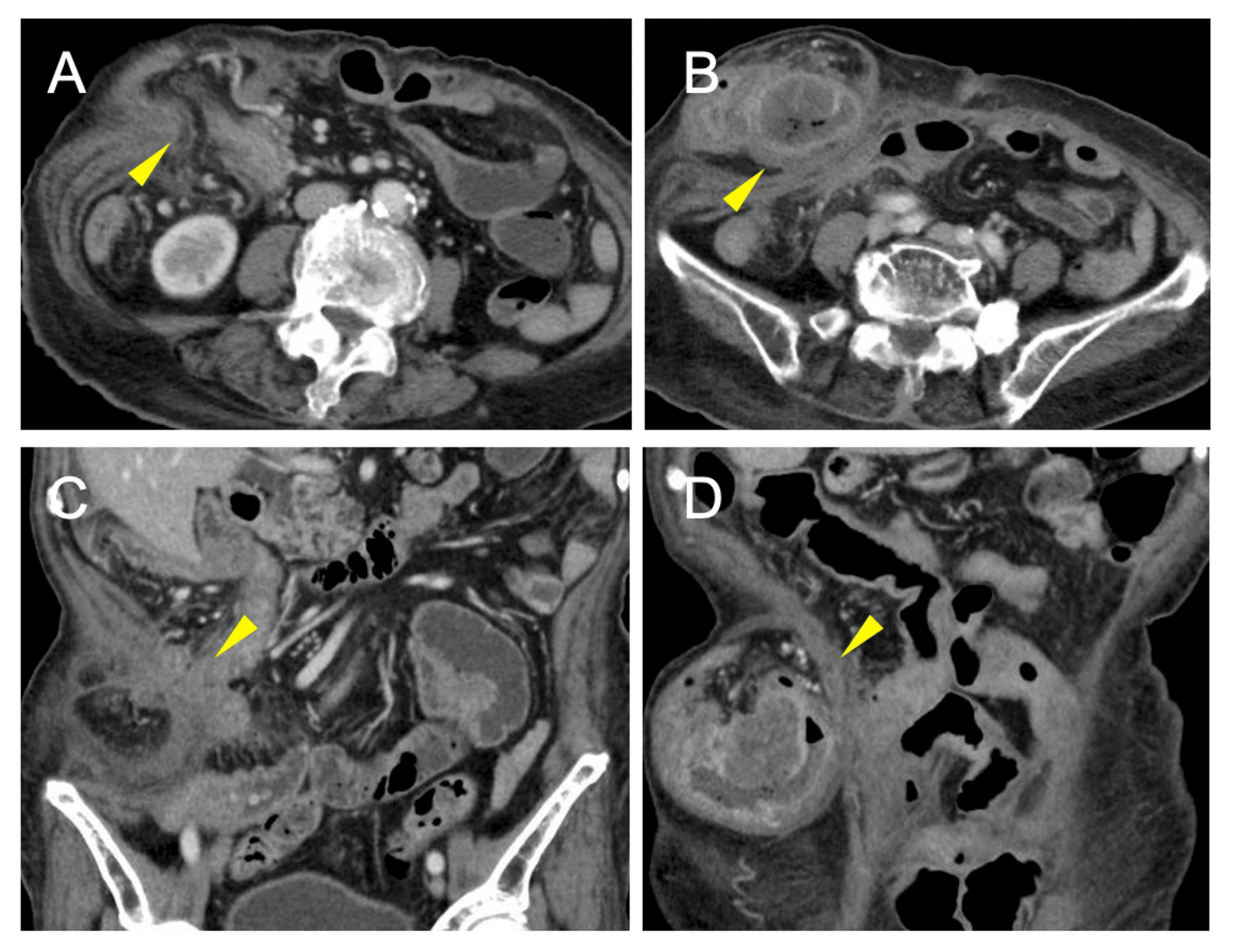
Contrast-enhanced CT. (A and B) Axial images showing an incisional hernia at the right subcostal region containing the ascending colon with wall thickening and diminished enhancement. (C and D) Coronal images showing the ascending colon herniating through the fascial defect with surrounding fat stranding.

Contrast-enhanced CT showed an incisional hernia at the right subcostal region containing the ascending colon with wall thickening, fat stranding, and diminished enhancement (Figure 1).

These findings suggested incarceration with venous congestion; an underlying tumor could not be excluded. No distant metastases or lymphadenopathy were seen.

Emergency laparotomy was performed. The ascending colon was incarcerated within the hernia sac at the cholecystectomy incision site and could not be reduced without enlarging the fascial defect. A hard mass at the cecum indicated malignancy. Right hemicolectomy with functional end-to-end anastomosis was performed. The hernia was closed primarily without mesh, given the contaminated field [7]. Operative time was three hours and 32 minutes.

**Figure 2:**
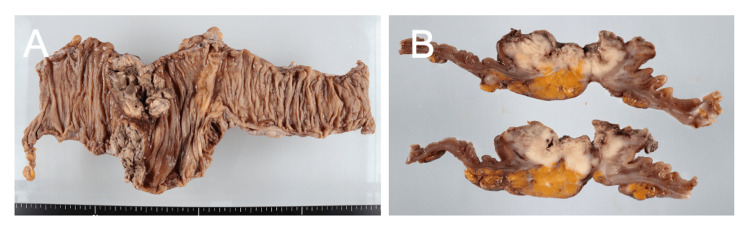
Macroscopic findings. (A) Resected specimen showing a 40 × 40 mm type 2 tumor at the cecum extending into the proximal ascending colon. (B) Cross-section showing transmural invasion to the serosa (pT4a).

Gross examination showed a 40 × 40 mm type 2 tumor at the cecum extending into the proximal ascending colon (Figure 2).

**Figure 3:**
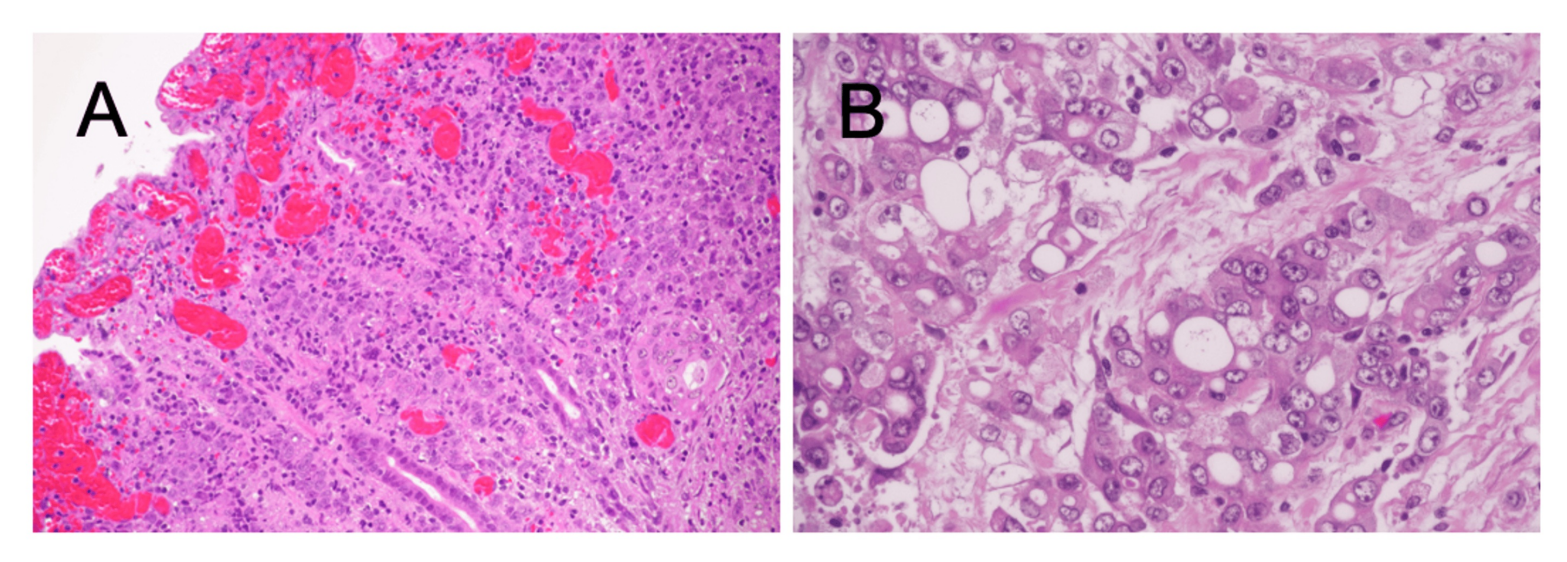
Histopathology (H&E). (A) Low-power view (original magnification: ×40) showing adenocarcinoma invading the serosa with venous congestion. (B) High-power view (original magnification: ×200) showing poorly differentiated adenocarcinoma. Lymphovascular invasion was limited (ly1 and v0), with no evidence of worsening by incarceration.

Histopathology revealed poorly to moderately differentiated adenocarcinoma (por1 > tub2, according to the Japanese Classification of Colorectal Carcinoma; por1: poorly differentiated adenocarcinoma, solid type; tub2: moderately differentiated tubular adenocarcinoma) invading the serosa (pT4a). Marked venous congestion was present, consistent with incarceration. Lymphovascular invasion was limited (ly1, minimal lymphatic invasion; v0, no venous invasion), with no evidence that incarceration had worsened tumor spread or caused rupture. All lymph nodes dissected were negative (pN0). Final staging was pT4aN0M0, stage IIB according to the Union for International Cancer Control (UICC)/American Joint Committee on Cancer (AJCC) eighth edition (Figure 3).

Postoperative recovery was uneventful. Oral intake began on day 2, and diet was advanced on day 7. Given the patient’s age (91 years) and node-negative status, adjuvant chemotherapy was not recommended [8,9]. She was discharged on postoperative day 22 and remained well at three-month follow-up.

## Discussion

This case describes colon adenocarcinoma arising from the cecum and extending into the proximal ascending colon, incarcerated within an incisional hernia, an entity that has not been previously reported in the literature. While colorectal cancer within inguinal hernias has been documented in approximately 40 cases worldwide, its occurrence within incisional hernias appears to be unreported [4-6]. This distinction is clinically significant: inguinal hernias arise through congenital or acquired defects in the inguinal canal, whereas incisional hernias develop at sites of previous surgical incisions, representing a distinct pathophysiological entity.

The existing literature on colorectal cancer within inguinal hernias provides useful context for understanding this rare clinical scenario. Chern et al. reviewed 38 cases and found that sigmoid colon involvement predominated (71.1%), with cecal cancer representing approximately 16% and ascending colon cancer only 5.2% of cases [4]. Zhang et al. confirmed these findings, reporting sigmoid involvement in 82.5% of cases in their systematic review [5]. The preponderance of sigmoid involvement likely reflects the greater mobility of this colonic segment compared to the retroperitoneal ascending colon.

Several anatomical factors may explain why our case is unique. First, the ascending colon, being retroperitoneal and relatively fixed, is less mobile than the sigmoid colon and therefore less prone to herniation of any type. Second, incisional hernias typically develop at midline incisions, whereas the right subcostal incision used for open cholecystectomy lies laterally, directly adjacent to the ascending colon. The anatomical proximity in our case may have facilitated the gradual incorporation of the colon into the hernia sac over the 30-year interval since the original surgery.

The pathophysiology underlying colorectal cancer within hernias remains unclear. Proposed mechanisms include malignant transformation occurring in chronically herniated bowel subjected to repeated mechanical stress and chronic inflammation or, alternatively, tumor growth causing decreased bowel mobility and subsequent incarceration [10,11]. Avidan et al. suggested that the association may be coincidental, reflecting the high prevalence of both conditions in elderly populations [12]. In our patient, distinguishing between these possibilities is not feasible.

Clinical presentation in reported inguinal hernia cases is variable. Zhang et al. reported that 47% presented with incarceration, 41% presented with perforation, and only 6% were uncomplicated [5]. Preoperative diagnosis is achieved in approximately half of cases [5]. Sabra et al. [13] and Mizuno et al. [14] reported cases where the diagnosis was made intraoperatively during emergency surgery for incarcerated inguinal hernias. Our patient presented atypically with gastrointestinal bleeding and anemia rather than symptoms directly referable to the hernia, highlighting the diagnostic challenge posed by this entity.

Surgical management must address both oncological and hernia-related considerations. Most reported cases have required emergency intervention through a combined approach [5,15]. R0 resection with appropriate lymphadenectomy should be pursued when feasible. Regarding hernia repair, the World Society of Emergency Surgery guidelines recommend caution with synthetic mesh in contaminated or potentially contaminated fields due to infection risk [7]. Primary fascial closure, as performed in our case, represents a reasonable approach despite higher recurrence rates, particularly in elderly patients with limited life expectancy [16].

An important consideration in managing incarcerated hernias containing colorectal cancer is the potential risk of tumor-related complications secondary to vascular compromise. In the present case, histopathological examination revealed marked venous congestion consistent with incarceration; however, there was no evidence of tumor rupture, cancer cell dissemination, or the exacerbation of lymphovascular invasion attributable to the incarcerated state. Nevertheless, the presence of significant venous congestion suggests that the incarcerated bowel segment was under considerable vascular stress, which could have led to tumor rupture or perforation if surgical intervention had been delayed. Such complications would substantially worsen the oncological prognosis by facilitating the peritoneal dissemination of cancer cells. Therefore, when encountering an incarcerated incisional hernia that is irreducible and malignancy cannot be excluded based on imaging findings, early surgical intervention should be planned without delay to prevent these potentially catastrophic complications.

The prognosis depends on tumor stage and the completeness of resection. Emergency presentation and advanced age contribute to perioperative risk. Despite these factors, our patient achieved R0 resection with a favorable short-term outcome. Given her age (91 years) and node-negative stage II disease, observation rather than adjuvant chemotherapy was elected. This decision was consistent with pooled analyses suggesting the limited benefit of adjuvant chemotherapy in very elderly patients, particularly those with stage II disease [8,9,17].

This case has several limitations. As a single case report, the findings cannot be generalized. Long-term follow-up data are not yet available. Additionally, while our literature search was comprehensive, we cannot exclude the possibility that isolated cases may exist in non-indexed journals or languages other than English and Japanese.

## Conclusions

To our knowledge, this is the first reported case of colorectal cancer incarcerated in an incisional hernia. This case demonstrates that colorectal malignancy should be considered in the differential diagnosis of complicated incisional hernias, especially in elderly patients with long-standing hernias or unexplained gastrointestinal symptoms. Preoperative imaging assists in surgical planning, and operative management should adhere to oncological principles while addressing the emergency nature of the presentation.
